# First-Time Usage of SGLT2 Inhibitors in Patients With Type 2 Diabetes Who Are Fasting Ramadan: Efficacy and Safety

**DOI:** 10.1155/jdr/4321423

**Published:** 2025-04-25

**Authors:** Hany Ahmed Muhammed Khalil, Sara Kasem Abdelal, Ahmed Faysal El-Rawy, Alshimaa Lotfy Hamed Abodahab

**Affiliations:** ^1^Internal Medicine Department, Faculty of Medicine, Sohag University, Sohag, Egypt; ^2^Cardiovascular Disease Department Faculty of Medicine, Sohag University, Sohag, Egypt

**Keywords:** dapagliflozin, efficacy, empagliflozin, Ramadan fasting, safety, SGLT2 inhibitors, Type 2 diabetes mellitus

## Abstract

**Introduction:** Ramadan fasting claims a necessary role in the management of diabetes. Many people with Type 2 diabetes insist on fasting during the holy month of Ramadan, which represents a challenge to their physicians to provide balance between preventing hypoglycemia or diabetic ketoacidosis (DKA) and good control of hyperglycemia with its short- and long-term complications. Sodium–glucose cotransporter 2 inhibitors (SGLT2is) are a glucose-lowering therapy for Type 2 diabetes, which are generally well tolerable but may carry the risk of dehydration and hypoglycemia particularly during the long fasting hours. The study aimed to assess the efficacy and safety of the use of SGLT2i for the first time during Ramadan fasting.

**Methods:** This prospective cohort study was carried out on 61 Egyptian Muslim patients, aged ≥ 18 years old, both sexes, with Type 2 diabetes mellitus (T2DM), prepared to fast during Ramadan, and treated with SGLT2i for the first time as a supplementary to metformin or another oral hypoglycemic drug. The dose of SGLT2i started after Iftar time. During and 6 weeks after Ramadan, evaluations were conducted.

**Results:** Glycated hemoglobin (HbA1c), blood pressure (systolic and diastolic), and creatinine were significantly lower after Ramadan than at the beginning of Ramadan. The estimated glomerular filtration rate (eGFR) was significantly higher after Ramadan than at the beginning of Ramadan. Hypoglycemia, dehydration, and DKA did not occur in any patient. There was a significant negative correlation between age and HbA1c (*r* = −0.267, 95% CI: −0.48 to −0.05; *p* = 0.037) and eGFR (*r* = −0.684, 95% CI: −0.79 to −0.54; *p* < 0.001) after Ramadan, while there was no correlation between the duration of DM and HbA1c before and after Ramadan. HbA1c was significantly lower after Ramadan than during Ramadan in patients with ischemic heart disease (IHD), hypertension (HTN), peripheral neuropathy (PN), and chronic kidney disease (CKD) (*p* < 0.05).

**Conclusions:** SGLT2i is effective and safe during Ramadan fasting with a significant reduction in HBA1c, blood pressure, and creatinine and a significant elevation of eGFR.

**Trial Registration:** ClinicalTrials.gov identifier: NCT06370247

**Summary**

• Why carry out this study?
  o. In 2019, globally, approximately, 460 million people were living with diabetes and, approximately, 116 million Muslim people who fast the holy month of Ramadan were living with diabetes, and this number is increasing.
  o. Is it safe to add SGLT2i for the first time during Ramadan fasting for better glycemic control?
  o. Lack of evidence and safety concerns about the beginning and continued use of SGLT2 inhibitor during Ramadan fasting indicated an increased risk of volume depletion, hypoglycemia, and risk of diabetic ketoacidosis.
• What was learned from the study?
  o. SGLT2i is effective and safe during Ramadan fasting with a significant reduction in blood pressure, HbA1c, and creatinine and a significant elevation of eGFR, and we did not detect any complications as dehydration, hypoglycemia, or diabetic ketoacidosis.
  o. The patients with comorbidities, for example, IHD, HTN, PN, and CKD, were the most beneficiary from addition of SGLT2i in control of diabetes, which needs further studies.

## 1. Introduction

Muslims worldwide observe a continuous fast from dawn to dusk in the holy month of Ramadan. Ramadan fasting must necessitate substantial alterations in the intake and frequency of meals. All Muslims who fast the holy month of Ramadan have metabolic changes as a result of these alterations [1].

Many Muslim patients with acute or chronic medical conditions prefer fasting. Religiosity, personal beliefs, and spirituality are crucial factors of the social determinants that impact health-related behaviors and treatment adherence of patients [2, 3].

Nevertheless, fasting is a deeply respected religious practice observed throughout the holy month of Ramadan, and devout Muslim individuals persist in fasting even when their healthcare doctors advise against it. In 2019, globally, approximately, 460 million people were living with diabetes and, approximately, 148 million Muslim people were living with diabetes, and this number is increasing [4]. It has been estimated that worldwide 116 million Muslim patients with diabetes fast; fasting can significantly affect blood glucose levels and overall metabolism. Careful management of patients with Type 2 diabetes mellitus (T2DM) need to control their condition during fasting to avoid acute complications like hypoglycemia or hyperglycemia [5].

Before Ramadan, several clinical studies, including those using insulin, glucagon-like peptide-1 agonists, sulphonylureas, and dipeptidyl peptidase inhibitors, evaluated the safety of pharmacological diabetic therapies [6–9].

SGLT2is represent a relatively new class of oral antidiabetic drugs that inhibit glucose reabsorption in the proximal tubule of the kidney, leading to glucosuria and subsequent reductions in blood glucose levels. Beyond their glucose-lowering effects, SGLT2is have demonstrated significant cardiovascular and renal benefits, making them a valuable therapeutic option for patients with T2DM, particularly those with comorbid conditions such as heart failure and chronic kidney disease (CKD) [10]. However, there are raised concerns regarding the potential risks of SGLT2i use during fasting, including volume depletion, dehydration, and an increased risk of diabetic ketoacidosis (DKA), particularly in the context of prolonged fasting hours during Ramadan [11].

Several studies have explored the safety and efficacy of SGLT2i during Ramadan fasting. For example, Bashier et al. conducted a study on Muslim patients with T2DM and found that SGLT2is were well tolerable during Ramadan, with no significant increase in adverse events such as hypoglycemia or DKA [12]. Similarly, Gad et al. performed a systematic review and meta-analysis, concluding that SGLT2i use during Ramadan was associated with improved glycemic control and no significant increase in adverse events compared to other antidiabetic agents [13]. However, other studies, such as those by Abdelgadir et al. and Sheikh et al., have highlighted the need for careful patient selection and monitoring when using SGLT2i during fasting, particularly in high-risk populations [14, 15].

Despite these findings, there remains a lack of robust evidence on the first-time use of SGLT2i during Ramadan fasting, particularly in patients with comorbid conditions such as hypertension (HTN), ischemic heart disease (IHD), and CKD. This gap in the literature underscores the need for further research to evaluate the safety and efficacy of initiating SGLT2i therapy during Ramadan, particularly in real-world clinical settings.

The study aimed to assess the efficacy and safety of the use of SGLT2i for the first time during Ramadan fasting. Therefore, we can give this drug without delay in Ramadan.

## 2. Methods

This prospective, cohort study was conducted on 61 Egyptian Muslim patients with T2DM, aged 18 years or older, of both sexes, with a confirmed diagnosis of T2DM (as per standard diagnostic criteria), and who intended to fast for at least 15 days during the holy month of Ramadan without any contraindication to fasting. Patients who were not previously on SGLT2 inhibitors (empagliflozin or dapagliflozin) and were initiated on these medications as an add-on to metformin or another oral hypoglycemic agent during Ramadan and who had stable medical conditions, including controlled HTN, IHD, CKD, and peripheral neuropathy (PN), were included.

The research was conducted during Ramadan 2023 after the ethical approval of the Sohag University Hospital ethical committee in Egypt (approval code: Soh-Med-23-4-07PD). Informed written consent was obtained from the patient. The study is in accordance with the Helsinki Declaration of 1964 and its later amendments.

The indications of adding SGLT2 inhibitors were first for better control of blood sugar and second for cardiorenal benefits especially in patients with ischemia or renal affection.

Exclusion criteria were chronic liver disease (aspartate aminotransferase (AST), alanine aminotransferase (ALT), or alkaline phosphatase blood levels over twice the normal value upper limit), CKD with estimated glomerular filtration rate (eGFR) of below 25 mL/min/1.73 m^2^, renal dialysis and ketoacidosis within the previous 6 months, prior diagnosis of cancer within the previous 3 years, strokes (including transient ischemic attack), and diabetic foot infection, nontraumatic minor amputation, or gangrene. In addition, pregnant or lactating women, individuals with a prior occurrence of recurrent urinary tract infections, and those who ceased taking SGLT2i throughout the holy month of Ramadan or fasted for less than 15 days were all excluded.

All patients were subjected to full history taking (duration of DM; current medication; other comorbid diseases like HTN, IHD, CKD, PN, dyslipidemia, and atherosclerotic cardiovascular diseases; the time of hypoglycemia; the number of symptomatic and recorded episodes of hypoglycemia; the number of days fasting; any hospitalizations required to treat hypoglycemia and hyperglycemia; and acute dehydration or DKA and any complaints of excessive and unusual thirst) and clinical assessment (weight, body mass index (BMI), systolic blood pressure (SBP), and diastolic blood pressure (DBP)).

We assessed HbA1c, creatinine, eGFR, urine analysis, and serum electrolytes (Na and K) during and after Ramadan, with reassessments after 6 weeks.

Sulfonylureas (SU) or other oral hypoglycemic medications were modified in observance of Ramadan, and SGLT2i dosing began after Iftar time. Diabetes education related to Ramadan was provided to participants by standard protocols. To minimize the impact of potential confounders, all patients received standardized diabetes education before Ramadan, which included guidance on dietary habits, physical activity, and medication adherence during fasting. However, we acknowledge that individual variations in these factors, such as differences in meal composition, exercise levels, and adherence to medication, could still influence the observed outcomes. Awareness and identification of warning indicators of hypoglycemia were assessed and reinforced during Ramadan evaluation. The dosage of SGLT2i (empagliflozin or dapagliflozin) and oral antidiabetic medicines were provided.

### 2.1. Statistical Analysis

SPSS V24 (IBM Inc., Chicago, IL, USA) was used for statistical analysis. The mean and standard deviation (SD) were used to represent quantitative variables, which were compared by the paired Student's *t*-test. The frequency and percentage were used to represent qualitative variables. The Pearson correlation was used to do the correlation between various variables. A two-tailed *p* value < 0.05 was considered statistically significant.

## 3. Results

The mean value (±SD) of age was 50.5 (±11.21) years. There were 25 (40.98%) male patients. The mean value (±SD) of the duration of DM was 5.8 (±4.9) years. HTN was present in 24 (39.34%) patients. IHD was present in 10 (16.39%) patients. CKD was present in 24 (39.34%) patients. PN was present in 24 (39.34%) patients. Thirty-three (54.1%) patients were receiving dapagliflozin, and 36 (59.02%) patients were receiving empagliflozin (Table 1).

Weight and BMI were insignificantly different between during Ramadan and after Ramadan. SBP was significantly lower after Ramadan than during Ramadan, with a mean reduction of −7.7 mmHg (95% CI: −10.2 to −5.2; Cohen's *d* = 0.52, moderate effect; *p* < 0.001). Similarly, DBP decreased significantly, with a mean reduction of −3.9 mmHg (95% CI: −5.1 to −2.7; Cohen's *d* = 0.58, moderate effect; *p* < 0.001) (Table 2).

HbA1c was significantly lower after Ramadan than during Ramadan, with a mean reduction of −0.5% (95% CI: −0.7 to −0.3; Cohen's *d* = 0.32, small effect; *p* < 0.001). Creatinine levels also decreased significantly, with a mean reduction of −0.1 mg/dL (95% CI: −0.15 to −0.05; Cohen's *d* = 0.45, small effect; *p* < 0.001). Conversely, eGFR was significantly higher after Ramadan, with a mean improvement of 5.6 mL/min (95% CI: 3.2–8.0; Cohen's *d* = 0.21, small effect; *p* < 0.001). Urine pus, Na, and K were insignificantly different during Ramadan and after Ramadan (Table 3).

There was a significant negative correlation between age and HbA1c after Ramadan (*r* = −0.267, 95% CI: −0.48 to −0.05; *p* = 0.037), as well as between age and eGFR during Ramadan (*r* = −0.667, 95% CI: −0.78 to −0.52; *p* < 0.001) and after Ramadan (*r* = −0.684, 95% CI: −0.79 to −0.54; *p* < 0.001). However, there was no significant correlation between age and HbA1c during Ramadan (*r* = −0.239, 95% CI: −0.45 to −0.02; *p* = 0.063). Additionally, there was no significant correlation between the duration of DM and HbA1c during Ramadan (*r* = 0.082, 95% CI: −0.18–0.34; *p* = 0.528) or after Ramadan (*r* = 0.0427, 95% CI: −0.22–0.30; *p* = 0.7439) (Table 4).

HbA1c was significantly lower after Ramadan than during Ramadan in patients with IHD (mean reduction: −0.6%, 95% CI: −1.0 to −0.2; Cohen's *d* = 0.43, small effect; *p* = 0.015), HTN (mean reduction: −0.5%, 95% CI: −0.8 to −0.2; Cohen's *d* = 0.30, small effect; *p* < 0.001), PN (mean reduction: −0.5%, 95% CI: −0.8 to −0.2; Cohen's *d* = 0.37, small effect; *p* < 0.001), and CKD (mean reduction: −0.6%, 95% CI: −1.2–0.0; Cohen's *d* = 0.29, small effect; *p* = 0.036). However, the delta change in HbA1c between patients with IHD, HTN, PN, and CKD was insignificantly different (*p* = 0.829) (Table 5).

Regarding safety, hypoglycemia, dehydration, and DKA did not occur in any patient.

## 4. Discussion

Diabetes management throughout the fasting month of Ramadan is a delicate and difficult matter. Indeed, the majority of Muslims with diabetes continue to fast despite medical advice to the contrary [11, 16]. However, there are significant hazards that fasting may entail [17].

Patients with diabetes who are at high or very high risk are susceptible to hypoglycemia (especially during the day), undetected hyperglycemia (especially at night), dehydration, and DKA (especially at night) during the fasting period of Ramadan [18]. Hence, it is critical to choose an antidiabetic drug that is supported by scientific data and does not typically induce hypoglycemia, which would need the patient to interrupt their fast [19].

It has been demonstrated that SGLT2i is safe and effective when used in conjunction with other oral hypoglycemic agents, metformin, and intensive insulin treatment in several randomized controlled trials [12, 20–22].

To the best of our knowledge, after intensive literature reading, this is the first study to assess the efficacy and safety of the first use of SGLT2i during Ramadan fasting. The existing studies primarily focus on using SGLT2i before Ramadan fasting [12, 14, 23].

Our study population, as shown in (Table 1), consisted of 61 patients with a mean age of 50.5 years, 40.98% of whom were male. The mean duration of diabetes was 5.8 years, and comorbidities such as HTN (39.34%), CKD (39.34%), and PN (39.34%) were prevalent. The majority of patients received either dapagliflozin (54.1%) or empagliflozin (45.9%). These demographic and clinical characteristics highlight the high-risk nature of the study population, which is representative of real-world patients with Type 2 diabetes fasting during Ramadan.

Our results revealed that the differences in weight and BMI between during Ramadan and after Ramadan were not statistically significant (Table 2). This lack of significant change in weight and BMI may be attributed to the short duration of the study or the specific population being studied, as SGLT2i is typically associated with weight loss over longer periods. In agreement with our results, Gad et al. [13] conducted a systematic review and meta-analysis that included five studies to evaluate the effects of Ramadan fasting on patients with T2DM treated with SGLT2i and showed that weight was insignificantly different between before Ramadan and after Ramadan.

In addition, our results supported Sheikh et al. [15] who noted that in T2DM using SGLT2i, the weight was insignificantly different between pre-Ramadan and after Ramadan.

Regarding weight and BMI, a study performed by Ahmed et al. [23] was consistent with our findings. Additionally, Pathan et al. [24] found similar results of weight in their study participants on empagliflozin. Moreover, Abdelgadir et al. [14] noticed that in T2DM using the SGLT2i group, weight was insignificant between before Ramadan and after Ramadan.

However, SBP and DBP were significantly lower after Ramadan, with mean reductions of −7.7 mmHg (95% CI: −10.2 to −5.2; Cohen's *d* = 0.52, moderate effect; *p* < 0.001) and −3.9 mmHg (95% CI: −5.1 to −2.7; Cohen's *d* = 0.58, moderate effect; *p* < 0.001), respectively. These findings are consistent with the known cardiovascular benefits of SGLT2i, which include blood pressure reduction and improved vascular function [15]. In contrary, Ahmed et al. [23] stated that SPB and DPB were insignificantly different between pre-Ramadan and after Ramadan. This discrepancy may be attributed to several factors. First, our study included a higher proportion of patients with comorbidities such as HTN and CKD, who may derive greater benefit from the cardiorenal effects of SGLT2 inhibitors. Second, our patients were initiating taking SGLT2 inhibitors for the first time during Ramadan and received standardized diabetes education, which may have enhanced adherence and blood pressure control. Third, regional and cultural differences in dietary habits and fasting practices between our Egyptian population and the population studied by Ahmed et al. could have influenced blood pressure outcomes. Finally, differences in study design, such as the timing and duration of follow-up, may also explain the varying results. Additionally, Abdelgadir et al. [14] noticed that SBP and DBP had insignificant differences between during Ramadan and after Ramadan in their study of high-risk insulin-treated patients using SGLT2 inhibitors. This difference may be attributed to the use of a flash glucose monitoring system in blood glucose level management and monitoring, which may have influenced patient behavior differently compared to our standard protocols.

Creatinine levels were significantly lower after Ramadan than during Ramadan, with mean reductions of −0.1 mg/dL (95% CI: −0.15 to −0.05; Cohen's *d* = 0.45, small effect; *p* < 0.001), respectively. Conversely, eGFR was significantly higher after Ramadan, with a mean improvement of 5.6 mL/min (95% CI: 3.2–8.0; Cohen's *d* = 0.21, small effect; *p* < 0.001) (Table 3). These findings suggest that SGLT2i not only improves glycemic control but also has beneficial effects on renal function during Ramadan fasting. In disagreement with our results, Sheikh et al. [15] noted that eGFR was significantly lower after Ramadan than pre-Ramadan. In addition, SBP, DBP, HbA1c, and creatinine were comparable after Ramadan to pre-Ramadan. The discrepancy in eGFR outcomes between the two studies may stem from differences in patient characteristics (e.g., baseline renal function and comorbidities such as HTN and CKD), study design (e.g., exclusion of patients with eGFR < 45 mL/min/1.73 m^2^ in Sheikh et al. versus inclusion of CKD patients in your study), and environmental factors (e.g., fasting duration, temperature, and hydration status during Ramadan). Sheikh et al. observed a decrease in eGFR, potentially due to transient dehydration, a healthier cohort, and the diuretic effects of SGLT2 inhibitors, while your study showed significant improvement in eGFR, likely reflecting the renoprotective benefits of SGLT2 inhibitors, particularly in patients with impaired renal function, as well as better management of hydration and comorbidities. Additionally, differences in concomitant medications, statistical methods, and follow-up timing may have contributed to the contrasting results.

However, Pathan et al. [24] found that creatinine and eGFR were insignificant between after Ramadan and before Ramadan. This difference may stem from differences in patient characteristics (e.g., inclusion of CKD patients in our study versus exclusion of eGFR < 45 mL/min/1.73 m^2^ in Pathan et al.'s study), study design (e.g., initiation of SGLT2 inhibitors during Ramadan in your study versus stable use in Pathan et al.'s study), and environmental factors (e.g., fasting duration and hydration status). Our study showed significant improvements in eGFR and reductions in creatinine, likely due to the renoprotective effects of SGLT2 inhibitors in patients with CKD and better hydration management, while Pathan et al.'s study found no significant changes, possibly due to stable medication use, healthier baseline renal function, and transient effects of fasting conditions. Differences in statistical methods, sample sizes, and follow-up timing may have also contributed to the contrasting results.

In contrast with our results, Abdelgadir et al. [14] found that creatinine and eGFR were higher after Ramadan than during Ramadan. This difference may be attributed to the use of a flash glucose monitoring system in blood glucose level management and monitoring, in which a sensor is applied on the upper arm. The sensor estimates glucose levels using the interstitial fluid under the skin and records accurate glucose levels 24 h a day. Every few minutes the sensor records glucose levels 24 h a day, giving more accurate results [14].

Interestingly, urine pus, Na, and K levels did not change significantly, which may be attributed to several factors, including stable hydration status due to adequate fluid intake during nonfasting hours, adherence to standardized diabetes education, renal adaptive mechanisms, the renal protective effects of SGLT2 inhibitors, balanced dietary habits during nonfasting periods, physiological adaptation to fasting, the exclusion of high-risk patients with recurrent UTIs or advanced CKD, and the relatively small sample size, all of which likely contributed to maintaining stable urine composition and electrolyte levels., as shown in Table 3.

Table 4 reveals no significant correlation between the duration of diabetes and HbA1c levels, either during or after Ramadan. This suggests that the duration of diabetes may not be a strong predictor of glycemic control in this cohort, possibly due to the heterogeneity of diabetes management strategies among patients with varying durations of diabetes.

Our results revealed that HbA1c levels were significantly lower after Ramadan than during Ramadan, with a mean reduction of −0.5% (95% CI: −0.7 to −0.3; Cohen's *d* = 0.32, small effect; *p* < 0.001). This reduction, though modest in magnitude, is clinically significant given the short duration of the study and the high-risk nature of the study population. Interestingly, for patients suffering from IHD, HTN, PN, and CKD, HbA1c was significantly lower after Ramadan than during Ramadan, with mean reductions of −0.6% (95% CI: −1.0 to −0.2; Cohen's *d* = 0.43, small effect; *p* = 0.015), −0.5% (95% CI: −0.8 to −0.2; Cohen's *d* = 0.30, small effect; *p* < 0.001), −0.5% (95% CI: −0.8 to −0.2; Cohen's *d* = 0.37, small effect; *p* < 0.001), and −0.6% (95% CI: −1.2–0.0; Cohen's *d* = 0.29, small effect; *p* = 0.036), respectively. However, the delta change in HbA1c between patients with different comorbidities was insignificantly different (*p* = 0.829), suggesting that the glucose-lowering effect of SGLT2 inhibitors is consistent across different patient subgroups.

In agreement with our results, Majid et al. conducted a systematic review and meta-analysis that included five studies to evaluate the effects of Ramadan fasting on patients with T2DM treated with SGLT2 inhibitors and showed that HbA1c was significantly lower after Ramadan than before Ramadan [17]. Similarly, Ahmed et al. found that HbA1c was lower after Ramadan than before Ramadan in their study of empagliflozin use during Ramadan [23]. However, Sheikh et al. noted that HbA1c was comparable after Ramadan to pre-Ramadan in their study of SGLT2 inhibitors during Ramadan fasting [15]. This discrepancy can be attributed to differences in study population characteristics (e.g., higher prevalence of comorbidities like HTN and CKD in our study, which may enhance the cardiorenal benefits of SGLT2 inhibitors), study design (our study focused on first-time SGLT2 inhibitor use during Ramadan, while Sheikh et al. included patients already on SGLT2 inhibitors), patient adherence (standardized diabetes education in our study may have improved adherence), regional and cultural factors (dietary habits and fasting practices in Egypt vs. Pakistan), and methodological differences (e.g., exclusion of patients with eGFR < 45 mL/min/1.73 m^2^ in Sheikh et al., which may have influenced renal and glycemic outcomes).

In the present study, there was a significant negative correlation between age and HbA1c after Ramadan (*r* = −0.267, 95% CI: −0.48 to −0.05; *p* = 0.037), eGFR during Ramadan (*r* = −0.667, 95% CI: −0.78 to −0.52; *p* < 0.001), and eGFR after Ramadan (*r* = −0.684, 95% CI: −0.79 to −0.54; *p* < 0.001).

HbA1c levels after Ramadan reflected the average blood glucose control over the previous 2–3 months, including the fasting month. The age at which patients begin using SGLT2i could reflect the duration and progression of their diabetes condition [25]. Older patients might have more prolonged exposure to high glucose levels and potentially more complications or a longer history of diabetes management strategies [26]. Starting SGLT2i later might suggest a switch or addition in therapy, possibly reflecting a more aggressive approach to controlling HbA1c levels that were not adequately managed with previous therapies. SGLT2i at an older age is associated with better after-Ramadan glycemic control [27–29]. Other several factors could be attributed to this finding: First, older individuals often have more experience managing their diabetes, leading to better adherence to medication and lifestyle modifications during Ramadan. Second, physiological changes associated with aging, such as more stable glucose metabolism, may contribute to improved glycemic control. Additionally, older patients may be more cautious about their dietary habits during fasting, avoiding excessive food intake during nonfasting hours (Iftar and Suhoor). Finally, the cardiorenal benefits of SGLT2 inhibitors may have a more pronounced effect in older patients with comorbidities, further enhancing glycemic outcomes. Ahmed et al. [23] found no significant correlation between age and HbA1c in their study of empagliflozin use during Ramadan. This difference may be due to the older age of our study population and the inclusion of patients with multiple comorbidities, who may benefit more from the cardiorenal effects of SGLT2 inhibitors.

While our study focused on Muslim patients fasting during Ramadan, the findings may also have implications for broader populations practicing other forms of fasting, such as intermittent fasting or time-restricted eating. The safety and efficacy of SGLT2 inhibitors during prolonged fasting periods, as observed in our study, suggest that these medications could be a viable option for individuals with Type 2 diabetes who engage in fasting for weight management or metabolic health. However, further researches are needed to confirm these findings in non-Ramadan fasting contexts and to explore the optimal use of SGLT2 inhibitors in populations practicing intermittent fasting. These efforts could help expand the clinical applications of SGLT2 inhibitors and improve diabetes management for a wider range of patients.

As limitations, first, the sample size of 61 participants is relatively small, which may limit the statistical power and generalizability of the findings. Second, the study was conducted at a single center in Egypt, which may restrict the applicability of the results to other populations with different ethnic, cultural, or healthcare settings. Third, the lack of a control group limits the ability to attribute effects solely to SGLT2i. However, the study reflects real-world practice during Ramadan fasting. Future controlled trials are needed for confirmation. Fourth, the 6-week follow-up period limits the assessment of long-term safety and efficacy of SGLT2i. Future studies with extended follow-up are needed to evaluate sustained outcomes. Fifth, this study did not explicitly account for potential confounders such as dietary changes, physical activity, or other medications, which could influence the observed outcomes. While standardized diabetes education was provided to minimize variability, individual differences in these factors may still affect the results. Future studies should address these factors to strengthen the conclusions.

## 5. Conclusions

SGLT2 inhibitors are effective and safe during Ramadan fasting, with significant reductions in HbA1c, blood pressure, and creatinine, as well as improvements in renal function. However, our study has limitations, including the lack of a control group, a short follow-up period, and a single-center design. Future research should include controlled trials with longer follow-up periods and diverse populations to confirm these findings and explore the long-term effects of SGLT2 inhibitors during Ramadan fasting. These efforts will help optimize diabetes management for Muslim patients with Type 2 diabetes.

## Figures and Tables

**Table 1:** Demographic data, medical history, and SGLT2i of the studied patients.

	***N*** **= 61**
Age (years)	50.5 ± 11.21
Sex	
Male	25 (40.98%)
Female	36 (59.02%)
Duration of DM (years)	5.8 ± 4.9
Medical history	
Hypertension	24 (39.34%)
IHD	10 (16.39%)
CKD	24 (39.34%)
PN	24 (39.34%)
Type of SGLT2i	
Dapagliflozin	33 (54.1%)
Empagliflozin	28 (45.9%)

*Note:* Data are presented as mean ± SD or frequency (%). SGLT2i: sodium–glucose cotransporter-2.

Abbreviations: CKD, chronic kidney disease; DM, diabetes mellitus; IHD, ischemic heart disease; PN, parental nutrition.

**Table 2:** Weight, BMI, and blood pressure of the studied patients.

	**During Ramadan (** **n** = 61**)**	**After Ramadan (** **n** = 61**)**	**Mean difference (95% CI)**	**Effect size (Cohen's ** **d** **)**	**p** ** value**
Weight (kg)	85 ± 15.49	84.8 ± 15.07	−0.2 (−0.5–0.1)	0.01 (trivial)	0.438
BMI (kg/m^2^)	30.5 ± 4.69	30.4 ± 4.64	−0.1 (−0.3–0.1)	0.02 (trivial)	0.523
Systolic blood pressure (mmHg)	131.9 ± 18.58	124.2 ± 10.57	−7.7 (−10.2 to −5.2)	0.52 (moderate)	< 0.001⁣^∗^
Diastolic blood pressure (mmHg)	78 ± 7.98	74.1 ± 5.28	−3.9 (−5.1 to −2.7)	0.58 (moderate)	< 0.001⁣^∗^

*Note:* Data are presented as mean ± SD.

Abbreviation: BMI, body mass index.

⁣^∗^Significant as *p* value ≤ 0.05.

**Table 3:** Laboratory investigation during Ramadan and post-Ramadan of the studied patients.

	**During Ramadan**	**After Ramadan**	**Mean difference (95% CI)**	**Effect size (Cohen's ** **d** **)**	**p** ** value**
HbA1c (%)	8.4 ± 1.68	7.9 ± 1.36	−0.5 (−0.7 to −0.3)	0.32 (small)	**< 0.001**⁣^∗^
Creatinine (mg/dL)	1.1 ± 0.23	1 ± 0.22	−0.1 (−0.15 to −0.05)	0.45 (small)	**< 0.001**⁣^∗^
eGFR (mL/min)	89.6 ± 26.14	95.2 ± 27.74	5.6 (3.2–8.0)	0.21 (small)	**< 0.001**⁣^∗^
Urine pus (pus cells)	5.9 ± 4.01	6.1 ± 4.71	0.2 (−0.5–0.9)	0.05 (trivial)	0.789
Serum Na (mEq/L)	138.7 ± 17.91	141.1 ± 2.64	2.4 (−2.1–6.9)	0.18 (trivial)	0.303
Serum K (mmol/L)	4.3 ± 0.5	4.2 ± 0.38	−0.1 (−0.2–0.0)	0.22 (small)	0.256

*Note:* Data are presented as mean ± SD. HbA1c: glycated hemoglobin.

Abbreviation: eGFR, estimated glomerular filtration rate.

⁣^∗^Significant as *p* value ≤ 0.05.

**Table 4:** Correlation between age and HbA1c and eGFR and between duration of DM and HbA1c of the studied patients.

			**Age (years)**
HbA1c (%)	During Ramadan	*r*	−0.239
		*p* value	0.063
		**95% confidence interval (CI)**	(−0.45 to −0.02)
	After Ramadan	*r*	−0.267
		*p* value	**0.037**⁣^∗^
		**95% confidence interval (CI)**	(−0.48 to −0.05)

eGFR (mg/dL)	During Ramadan	*r*	−0.667
		*p* value	**<0.001**⁣^∗^
		**95% confidence interval (CI)**	(−0.78 to −0.52)
	After Ramadan	*r*	−0.684
		*p* value	**<0.001**⁣^∗^
		**95% confidence interval (CI)**	(−0.79 to −0.54)

			**HbA1c (%)**

Duration of DM (years)	During Ramadan	*r*	0.082
		*p* value	0.528
		**95% confidence interval (CI)**	(−0.18–0.34)
	After Ramadan	*r*	0.0427
		*p* value	0.7439
		**95% confidence interval (CI)**	(−0.22–0.30)

*Note:r*: Pearson's coefficient. HbA1c: glycated hemoglobin.

Abbreviations: eGFR, estimated glomerular filtration rate; DM, diabetes mellitus.

⁣^∗^Significant as *p* value ≤ 0.05.

**Table 5:** Relation between ischemic heart disease, hypertension, and PN and HbA1c of the studied patients.

	**During Ramadan**	**After Ramadan**	**Mean difference (95% CI)**	**Effect size (Cohen's ** **d** **)**	**p** ** value #**	**Delta change**
IHD (*n* = 10)	8.4 ± 1.53	7.8 ± 1.11	−0.6 (−1.0 to −0.2)	0.43 (small)	**0.015**⁣^∗^	−0.6 ± 0.59
Hypertensive (*n* = 24)	8.4 ± 1.84	7.9 ± 1.53	−0.5 (−0.8 to −0.2)	0.30 (small)	**< 0.001**⁣^∗^	−0.4 ± 0.5
PN (*n* = 24)	8.4 ± 1.44	7.9 ± 1.21	−0.5 (−0.8 to −0.2)	0.37 (small)	**< 0.001**⁣^∗^	−0.5 ± 0.41
CKD (*n* = 4)	8.5 ± 2.18	7.9 ± 1.95	−0.6 (−1.2–0.0)	0.29 (small)	**0.036**⁣^∗^	−0.6 ± 0.32
*p* value ##	1	0.996			—	0.829

*Note:* Data are presented as mean ± SD HbA1c: hemoglobin A1c. CKD: chronic kidney disease. *p* value #: between during Ramadan and after Ramadan assessment. *p* value ##: between groups.

Abbreviations: IHD: ischemic heart disease. PN: peripheral neuropathy.

⁣^∗^Significant as *p* value ≤ 0.05.

## Data Availability

The authors have nothing to report.
